# Association Between Adverse Childhood Experiences, Resilience and Mental Health in a Hispanic Community

**DOI:** 10.1007/s40653-022-00437-6

**Published:** 2022-01-22

**Authors:** Maribel G. Dominguez, Louis D. Brown

**Affiliations:** 1 Department of Public Health, University of Texas at El Paso, P.O. Box 960581, El Paso, Texas, 79996 USA; 2 Department of Health Promotion and Behavioral Sciences, School of Public Health in El Paso, The University of Texas Health Science Center at Houston, Texas, US

**Keywords:** Adverse childhood experiences (ACE), Mental distress, Resilience, Stressful life events, Trauma

## Abstract

This study explores the relations between adverse childhood experiences (ACEs), mental health and resilience among Hispanic adults living in the United States – Mexico Border region. Numerous studies have investigated the negative impact of ACEs on adult mental health, but the concept of resilience as a protective factor for mental health in the Hispanic communities has limited consideration in ACE treatment interventions. The proposed study addresses this gap in knowledge by investigating relations between ACEs, resilience, and mental health. An online survey was administered to 221 university students to assess the relationship between ACEs, mental distress and resilience. Using hierarchical linear regression, three models were estimated. First, including demographics, second including ACEs and low resilience, followed by the interaction of ACEs and resilience. Analyses indicate that ACEs were associated with mental distress (B = 1.02, 95% CI 0.37 – 1.68, *p <* 0.01) and low resilience was associated with mental distress (B = 5.37, 95% CI 3.15 – 7.59, *p <* .01). The interaction between ACEs and low resilience was also related to mental distress (B = 1.32, 95% CI 0.17 – 2.47, *p =* 0.03), indicating that ACEs had a larger association with mental distress among respondents with low resilience. Findings highlight the importance of the direct association between resilience and mental distress, along with the moderating influence of resilience on the relation between ACEs and mental health. Interventions promoting resilience may be effective in reducing mental distress, especially among individuals with a history of ACEs.

Extensive research demonstrates that individuals exposed to ACEs present higher rates of anxiety, depression, and suicide attempts, among other mental and physical health issues (Agorastos et al., 2014; Chang et al., 2019; Edward, 2003; Karatekin, 2018). Resilience is a promising protective factor that may help reduce the influence of ACEs on mental health by helping individuals overcome adversity (Hamby et al., 2018; Ungar, 2013; Venta et al., 2019). Yet little empirical work investigates the relationship between ACEs, mental health and resilience, in Hispanic communities (Newcomb et al., 2003; Merino et al., 2017). This study seeks to fill this gap in the literature by examining the relationship between ACEs, mental distress, and resilience among adults in a unique and predominantly Hispanic population on the U.S.—Mexico border.

## Adverse Childhood Experiences (ACEs)

ACEs include psychological, physical, and sexual abuse, neglect and household dysfunction including domestic violence, divorce, parental incarceration and substance abuse (Felitti et al., 1998). One explanation for the impact ACEs have on health is through neurodevelopmental changes (Brown et al., 2013; Navalta et al., 2018). A child exposed to toxic stress may experience unusual wear and tear on their neurophysiological regulation state (Sciaraffa et al., 2018). The child’s vulnerability to developing biological, psychological, behavioral and mental health issues in adulthood has then increased, specifically because they are at a vulnerable developmental stage (Johnson et al., 2013).

A dose–response relationship was found between ACE’s and the leading causes of death in the United States (Felitti et al., 1998). At least six in ten people in the general population have been exposed to at least one type of ACE (Brown et al., 2013). ACE prevalence recorded by 23 U.S. States using the Behavioral Risk Factor Surveillance System from 2011 – 2014 was 15.8% for exposure to four or more ACEs, and 45.7% for exposure to one or more ACEs (Merrick et al., 2018).

## ACEs, Mental Health and Mental Distress

Mental health is defined as the state of wellbeing where an individual is able to cope effectively with normal stresses, work productively and contribute to their own community while realizing their own potential (WHO, 2004). In contrast to mental health, mental distress causes pathological symptoms and diagnoses that range in severity and that may have lifelong impacts on health and disease risk (Hammarström, 2016; Prince et al., 2016). Mental distress is a concept used to define a painful set of mental symptoms that affect the normal state of mood in individuals (American Psychological Association, 2020).

ACEs have been associated with the development of psychiatric disorders (Elkins et al., 2019). For example, Agorastos et al., (2014) found that having a single ACE was a risk factor for the development of depression (OR: 2.2, 95% CI: 1.3—3.8). A dose–response relationship between ACE scores and depressive disorders has also been documented, with a 23% lifetime prevalence of depressive disorders among individuals experiencing one or more ACEs (Mersky et al., 2013; Poole et al., 2017). Further, the consequences of ACEs may be more severe in Hispanic communities, as previous research found larger associations between ACEs and chronic disease among Hispanic relative to non – Hispanic adolescents (Elkins et al., 2019). Other studies have shown that Hispanic respondents present higher ACEs and higher levels of mental distress, less peace, and more nervousness (Barrera et al., 2019; La Brenz et al., 2020; Zetino et al. (2020).

## Resilience as a Protective factor

This study conceptualizes resilience as a personal quality that allows someone to overcome adversity, through the use of internal resources such as self-esteem, strength, humor, problem solving, stress management, and adaptability to change (Connor & Davidson, 2003). We do not include in our conceptualization of resilience external resources that may also help an individual cope with adversity. We focus on resilience as an internal resource because it may differentiate between the success of individuals facing similarly challenging external circumstances in an under-resourced Hispanic community. Resilient coping processes serve as a protective strategy following adverse events and are associated with positive development under stress (Forbes et al., 2018; Spencer-Hwang et al., 2018; Ungar & Liebenberg, 2011). These internal characteristics are changeable and previous research suggests they can be cultivated (Martel et al., 2007; Nurius et al., 2020a, b). Interventions addressing resilience have improved personal confidence, socials skills and self-efficacy (Bray & Swann, 2018; Mackay et al., 2017).

Although our conceptualization of resilience is widespread in the literature, previous studies on ACEs, resilience, and mental health have varied in their operationalization of resilience. Specifically, one study found that the association between ACEs and adult mental distress was substantially weaker among individuals who always had a trusted adult available as a child (Bellis et al., 2017). Another study found the association between ACEs and poor mental health was lower among individuals with the resilience resources of adequate sleep, social support, and life satisfaction (Logan – Greene et al., 2014). Thus, previous research suggests resilience moderates the relationship between ACEs and mental distress, but it remains unclear how the operationalization of resilience may impact its moderating effect.

Furthermore, there is no literature to our knowledge exploring the role of resilience in understanding relations between ACEs and mental health in Hispanic communities such as the U.S.-Mexico border region. Understanding resilience as it relates to ACEs and mental health can contribute to the development of trauma informed interventions building resilience as a modifiable protective factor to help alleviate the effects of ACEs (Logan – Greene et al., 2014; Purewal et al., 2016). Disparities in the provision of mental health care are particularly severe for vulnerable Hispanic populations (Liu et al., 2020; Venta et al., 2019; Villalobos & Bridges, 2018). The high rates of poverty and limited access to health insurance inhibit access to mental health treatment (Caballero et al., 2017). This study may inform the development of resilience-building interventions, a potentially cost-effective strategy for alleviating the deleterious effects of ACEs in Hispanic communities.

## The Present Study

**Fig. 1:**
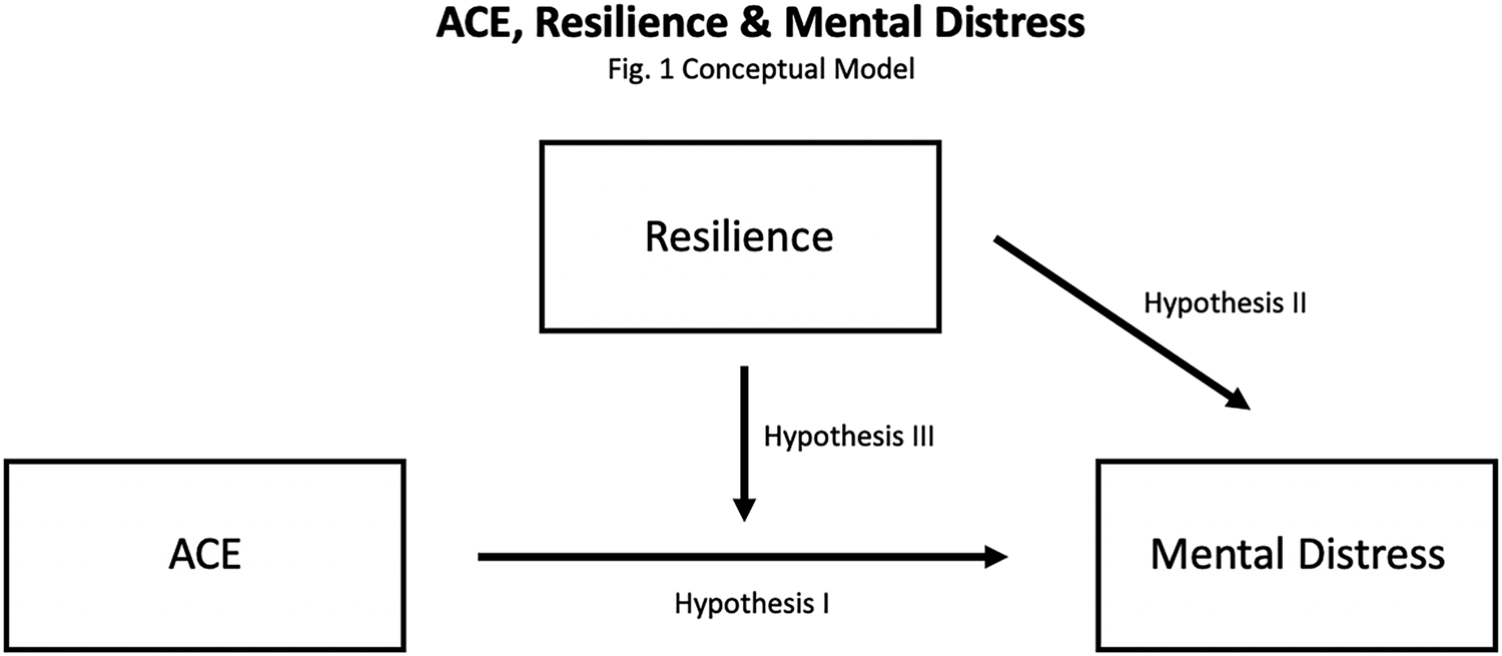
Conceptual Framework

Although extensive literature links ACEs with mental health, it is unclear how the resilience of individuals exposed to ACEs may alter the connection between ACEs and mental health, especially in Hispanic communities. Thus, the goal of this study is to examine relations between ACEs, mental health, and resilience in a Hispanic community. Based on previous research, our conceptual framework (see Fig. 1) describes how ACEs prevent individuals from having optimal health, and how resilience can play a protective role against the development of poor mental health.

The aim of the study is to test this conceptual model based on the following hypotheses:

1. ACEs are positively related to mental distress
2. Resilience is negatively associated with mental distress
3. The positive relation between ACEs and mental distress only exists in the lower resilience population.

## Methods

This study was reviewed and approved by the University of Texas at El Paso Institutional Review Board on March 12, 2019.

### Participants

Participants were English and Spanish speaking students from the University of Texas at El Paso, an institution with an 80% Hispanic population (UTEP CIERP, 2018). A total of 221 participants answered the survey. Inclusion criteria required for participants to be current students and be at least 18 years of age. Students from all degree programs including undergraduate and graduate were allowed to participate. A target sample size of 220 was determined through an a priori power analysis, which allowed us to detect a population correlation of 0.2 with a power of 0.08 with a p value of 0.05 (Cohen, 1988).

### Procedures

A 15-min-long anonymous electronic survey was administered to participants using the Question Pro Platform. The survey had an initial landing page that included the consent form, once participants agreed to participate the electronic survey would commence, An opportunistic sampling technique was used for recruitment. The recruitment strategy was to distribute wallet size flyers in both English and Spanish with a QR code and concise instructions that directed participants to the survey. Flyers were distributed after three undergraduate classroom announcements along with two visits to the student union, four visits to the library, and four visits to the student food court.

### Measures

This study used a quantitative cross-sectional design. A survey was assembled with scales that measured each of the three main constructs, along with sociodemographic characteristics and past year threatening events. The study questionnaire was constructed from valid and reliable measures of ACEs, mental distress and resilience in both English and Spanish (Labrenz et al., 2019; Soler Sánchez et al., 2016; Zhang et al., 2012).

### Demographic

Demographic questions detailed in Table 1, assessed age, biological sex, Hispanic or Latino origin, race/ethnicity, employment level, income, marital status, place of birth and education status.

**Table 1:** Demographic Information

**Characteristics**		**N**	**%**	**Mean**	**SD**
**Sample Size**		**221**			
Biological Sex	Female	147	66.5	1.67	0.47
	Male	74	33.5		
Place of Birth	Not U.S. Born	62	28.1	0.72	0.45
	U.S. Born	159	71.9		
Ethnicity	Non-Hispanic	48	21.7	0.78	0.41
	Hispanic	173	78.3		
Employment	Not Employed	29	13.1	1.56	1.61
	Employed	154	69.7		
	No Response	38	17.2		
Income	Less than 20,000	175	79.2	1.3	0.71
	20,000 to 40,000	28	12.7		
	40,000 to 60 000	11	5		
	60,000 to 70,000	1	0.5		
	70,000 or more	3	1.4		
Marital Status	Married	12	5.4	1.95	0.23
	Not Married	209	94.6		
Education	High school graduate	31	14	3.13	0.72
	Some college	141	63.8		
	Bachelor’s Degree	42	19		
	Master Degree	4	1.8		
	Doctorate Degree	3	1.4		

### Adverse Childhood Experiences (ACE) Questionnaire

The ACE questionnaire is a 10-item scale developed by researchers from the Kaiser Permanente’s San Diego Health Appraisal Clinic and the CDC (Felitti et al., 1998). The questionnaire assessed childhood adversity and includes exposures to household dysfunction, neglect, physical, sexual and emotional abuse (Felitti et al., 1998). Following Felitti et al. (1998), the total ACE score was computed by summing the “yes” responses to childhood exposures.

### Connor—Davidson Resilience Scale (CD-RISC 10)

The scale measures resilience as the ability to thrive in the face of adversity, assessing self-esteem strength, humor, problem solving, successfully coping with stress, and adaptability to change (Connor & Davidson, 2003). The CD – RISC scale is widely utilized because of its validity and reliability in different countries and languages (Connor & Davidson, 2003). The CD-RISC is a 10 item Likert scale, with answer options “Not True at all”, “Rarely True”, “Sometimes True”, “Often True” and “True nearly all the time.” A higher score represented higher resilience levels. In this study the scale had a good internal consistency (α = 0.86).

### Kessler Psychological Distress Scale (K10)

The Kessler Psychological Distress Scale (K10) is a 10-item questionnaire measuring psychological distress based on questions regarding symptoms of anxiety and depression (Prochaska et al., 2012). The K10 is a widely used and well validated scale that assesses the psychological distress among clinical and general populations (Cronbach alpha = 0.88) (Easton et al., 2017). The K10 scale has a five-level response scale, “All of the time”, “Most of the time”, “Some of the time”, “A little of the time” and “None of the time” with a range of a low 10 to a high 50 (Kessler et al., 2003). The scale demonstrated high reliability in this study (α = 0.93).

### Past Year Threatening Events

To differentiate ACEs from more recent adverse experiences, we assessed past year threatening events with the List of Threatening Experiences (LTE) scale. This 12-item scale developed by Motrico et al. (2013) has high sensitivity and reliability (Kappa range = 0.61– 0.87). Each item depicts a life event that has been identified as inducing stressful psychological experiences. The scale included the main question “Has any of the following events have occurred to you in the last 6 months?” related to various responses including financial crisis, serious illness or relationship problems. Each yes response to an LTE accounted for a value of 1, with scores ranging from 0 – 12. Internal consistency was modest, which is typical of count measures (α = 0.60).

### Data Analyses

In order to test all three hypotheses, we used hierarchical linear regression, with mental distress as the dependent variable. In the first model, we entered the demographic characteristics and past year threatening events, which served as covariates in our model. In the second model we tested the first and second hypotheses by adding ACEs and resilience to the model. In the third model we tested the third hypotheses by adding the ACEs x resilience interaction term to the model. All analyses were conducted using IBM SPSS Statistics 25. We also analyzed the data with non-Hispanics excluded, reaching identical conclusions on our hypothesis tests. We report findings from only the full sample because it offers more precise estimates of the relations under investigation.

## Results

### Sample Demographics

A total of 221 participants completed the survey, with all participants completing all survey items under investigation. Table 1 reports the demographic characteristics of participants in detail. Respondents were 67% female, with 72% born in the United States, 78% of Hispanic descent, and 80% with yearly incomes of less than $20,000. The survey was available in English and Spanish 89.7% of participants took the English version and thus 10.3% the Spanish version.

Table 2 presents the covariates and model tested hierarchical regression model. Step one of the hierarchical regression model predicting mental distress entered these sociodemographic covariates, along with past year threating events. The only significant sociodemographic characteristic was being U.S. born, which was associated with higher mental distress (B = 2.75, [0.01, 5.40], *p =* 0.05). Past year threatening events also had a significant and positive association with mental distress (B = 1.87, [1.16, 2.64], *p <* 0.01). All covariates together accounted for 15.2% of the variance in mental distress.

**Table 2:** Regression models predicting Mental Distress with Demographic Characteristics, Past Year Threatening Events (LTE), Adverse Childhood Experiences (ACEs), Low Resilience, and ACEs x Resilience

**Model 1**	**B**	95% CI	**β**	95% CI	Sig	${{\varvec{R}}}^{2}$	${\Delta {\varvec{R}}}^{2}$
Intercept	**14.02**	[2.74, 29.46]			**0.08**	0.39	0.15
Gender^1^	1.00	[-1.48, 3.48]	0.05	[-0.08, 0.18]	0.43		
Place of Birth^2^	**2.75**	[0.1, 5.40]	0.13	[0.01, 0.27]	**0.04**		
Ethnicity^3^	-0.18	[-3.09, 2.74]	-0.01	[-0.14, 0.12]	0.91		
Employment^4^	-0.55	[-1.29, 0.20]	-0.09	[-0.23, 0.04]	0.15		
Income^5^	0.04	[-1.73, 1.79]	0.00	[-0.13, 0.14]	0.97		
Marital Status^6^	0.59	[-5.33, 6.50]	0.02	[-0.13, 0.16]	0.85		
Education^7^	1.04	[-0.77, 2.86]	0.08	[-0.06, 0.22]	0.26		
Past Year Threatening Events	**1.87**	[1.16, 2.59]	0.34	[0.21, 0.47]	** < 0.01**		
**Model 2**						0.51	0.11
Intercept	7.75	[-6.86, 22.37]			0.30		
Gender^1^	0.88	[-1.44, 3.20]	0.05	[-0.08, 0.17]	0.46		
Place of Birth^**2**^	**3.55**	[1.05, 6.05]	0.17	[0.05, 0.30]	**0.01**		
Ethnicity^3^	-0.21	[-2.94, 2.52]	-0.01	[-0.13, 0.11]	0.88		
Employment^4^	-0.41	[-1.11, 0.29]	-0.07	[-0.19, 0.05]	0.25		
Income^5^	0.13	[-1.53, 1.80]	0.01	[-0.12, 0.14]	0.88		
Marital Status^6^	1.92	[-3.64, 7.48]	0.05	[-0.09, 0.19]	0.50		
Education^7^	1.02	[-0.68, 2.72]	0.08	[-0.05, 0.21]	0.24		
Past Year Threatening Events	**1.29**	[0.55, 2.03]	0.23	[0.1, 0.37]	** < 0.01**		
Adverse Childhood Experiences (ACEs)	**1.02**	[0.37, 1.68]	0.21	[0.08, 0.35]	** < 0.01**		
Low Resilience	**5.37**	[3.15, 7.59]	0.29	[0.17, 0.41]	** < 0.01**		
**Model 3**						0.53	0.18
Intercept	10.80	[-3.92, 25.51]			0.15		
Gender^1^	0.51	[-1.81, 2.83]	0.03	[-0.09, 0.15]	0.67		
Place of Birth^**2**^	**3.81**	[1.32, 6.29]	0.19	[0.07, 0.31]	** < 0.01**		
Ethnicity^3^	-0.25	[-2.95, 2.46]	-0.01	[-0.13, 0.11]	0.86		
Employment^4^	-0.37	[-1.06, 0.32]	-0.06	[-0.19, 0.06]	0.29		
Income^5^	0.09	[-1.56, 1.74]	0.01	[-0.12, 0.14]	0.92		
Marital Status^6^	1.27	[-4.27, 6.80]	0.03	[-0.11, 0.17]	0.65		
Education^7^	0.94	[-0.75, 2.63]	0.07	[-0.06, 0.21]	0.27		
Past Year Threatening Events	**1.19**	[0.45, 1.93]	0.21	[0.08, 0.35]	** < 0.01**		
Adverse Childhood Experiences (ACEs)	0.48	[-0.32, 1.28]	0.10	[-0.07, 0.27]	0.23		
Low Resilience	**3.11**	[0.16, 6.07]	0.17	[0.01, 0.33]	**0.04**		
ACEs x Low Resilience	**1.32**	[0.17, 2.47]	0.22	[0.03, 0.41]	**0.03**		

^1^Male = 1, Female 2; ^2^Not—U.S. Born = 0, U.S. Born = 1; ^3^Non—Hispanic = 0, Hispanic = 1; ^4^Not Employed = 0, Employed = 1; ^5^20,000 or less = 1, 20,000—40,000 = 2, 40,000—60,000 = 3, 60,000—70,000 = 4, 70,000 or more = 5; ^6^Not Married = 0, Married = 1; ^**7**^No High School = 1, High School = 2, Some College = 3, Bachelors = 4, Masters = 5, Doctorate = 6

### Hypothesis Tests

#### Hypothesis 1: ACE’s are Positively Related to Mental Distress

Our first hypothesis was tested in model 2. ACEs were positively associated with mental distress, consistent with our first hypothesis (B = 1.02, [0.37, 1.68], *p <* 0.01). An increase of one standard deviation in ACEs, predicted a 0.21 standard deviation increase in mental distress. Model 2 included ACEs and low resilience, accounting for 26.4% of the variance in mental distress, as compared to 15.2% in model 1, which just had the covariates.

#### Hypothesis 2: Resilience is Negatively Associated with Mental Distress

Consistent with our second hypothesis, mental distress was associated with low resilience (B = 5.37 [3.14, 7.60], *p <* 0.01). Low resilience was determined by the calculation of scores less than or equal to 29 total points. The model predicted 0.29 standard deviation increase in mental distress for respondents with low resilience (CI = [0.17, 0.41], *p <* 0.01).

#### Hypothesis 3: The Positive Relation Between ACEs and Mental Distress only Exists in the Lower Resilience Population

**Fig. 2:**
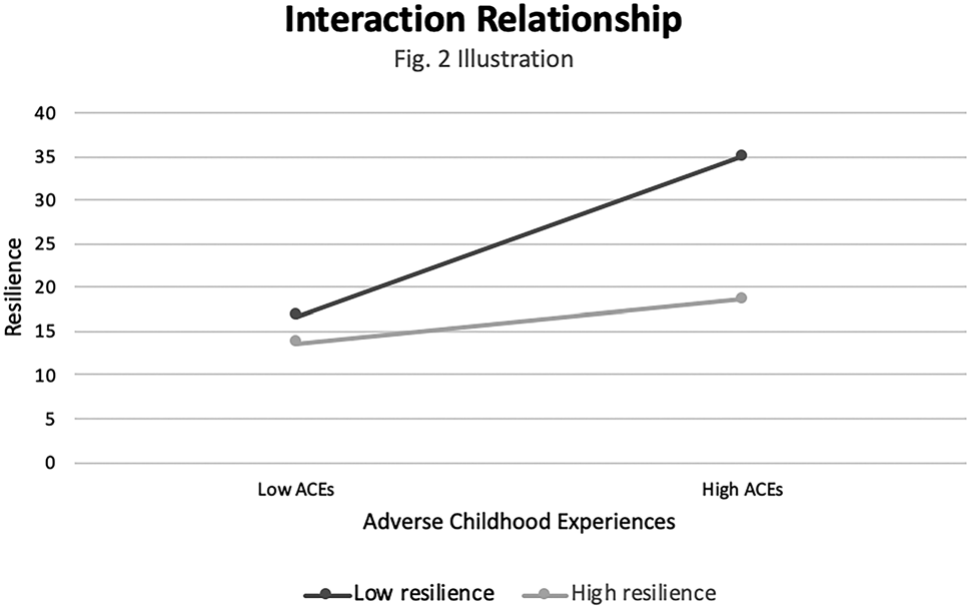
Interaction Relationship: Adverse Childhood Experiences (ACEs) and Resilience

As illustrated by Fig. 2, the results from our third model were also consistent with our third hypothesis; the ACEs x Low Resilience interaction term was significantly and positively associated with mental distress (B = 1.32, [0.17, 2.47], *p =* 0.03). Thus, among low resilience participants (but not high resilience participants), a one standard deviation increases in ACEs predicted a 0.23 standard deviation increase in mental distress. Further, the relation between ACEs and mental distress independent of resilience status decreased from B = 1.02 ([0.37, 1.68], *p <* 0.01) in Model 2 to a non-significant value of B = 0.48 ([-0.32, 1.28], *p =* 0.23) in Model 3, due to the addition of the ACEs x Low Resilience interaction term. The third and final model accounted for 28.2% of the variance of mental distress (R^2 =^ 0.28), relative to 26.4% of the variance in model 2.

## Discussion

This study examined the understudied role of resilience in understanding the relation between ACEs and mental distress in a Hispanic community. Each of the three core hypotheses were supported by our findings. Specifically, ACEs and resilience were related to mental distress, with resilience moderating the relation between ACEs and mental distress. These findings highlight the importance of cultivating resilience to mitigate the influence of ACEs on mental distress. We consider the implications of each of the three core hypotheses in more detail here.

Our first hypothesis, that ACEs are positively associated with mental distress, was supported by our data and previous research (Bellis et al., 2017; Easton, 2017; Felitti et al., 1998; Manyema et al., 2018; Martín-Blanco et al., 2016; Logan – Greene et al., 2014). The association between ACEs and mental distress may follow from neurophysiological dysregulations created by ACEs, which operate as stressors that dysregulate a child’s development. As a consequence, adverse experiences create cognitive, social, physical, behavioral and mental challenges linked to chronic diseases and risk behaviors (Felitti et al., 1998; Gilbert et al., 2015). Based on this finding it is not only important to prevent ACEs but also to help people recover from their effects, as research demonstrates that the impact of ACEs on adult mental health are severe and long-lasting (Felitti et al., 1998).

Our second hypothesis, that mental distress is associated with low resilience, was also supported by our findings. Consistent with previous research, resilience plays a protective role in mental health. Literature suggests that resilience can help people overcome the effects of life adversities and regulate mental health symptoms, thereby promoting psychological endurance (Banyard et al., 2017; Rutten et al., 2013). Thus, higher rates of resilience have been associated with less disability, better self-reported health, emotional equilibrium, and improved quality of life (Robottom et al., 2011). Due to the role that resilience plays in overcoming adversity, promoting resilience can help improve mental health and thus alleviate the effects of ACEs, allowing for a better quality of life after adversity. Research suggests resilience is a changeable target for interventions, as strategies that enhance resilience are emerging (Bray & Swann, 2018; Mackay et al., 2017; Martel et al., 2007; Nurius et al., 2020a, b).

The third hypothesis, that the relation between ACEs and mental distress only exists in the lower resilience population, was also supported. These findings are consistent with previous research (Bellis et al., 2017; Logan Greene et al., 2014) but unique in that resilience is defined more broadly in the current study. The current study utilized a self-rated resilience scale with reliable psychometric properties that allowed participants to select their own degree of agreement with each resilience item. Our findings highlight the importance of resilience in mitigating the severe consequences of ACEs on health and well-being. Given the prevalence of ACEs in our society, the cultivation of resilience may provide substantial health benefits. The basis of this finding lies in early life experiences, which set the path for adult mental health, with resilience helping people cope with early life adversity (as illustrated in Fig. 1).

In comparison to previous ACE studies, our study worked with a sample of predominantly Hispanic (78%) individuals of which 80% were low-income status. As reported in Table 3, participants reported relatively high rates of ACES, with 16% reporting exposure to four or more ACEs and 67% reporting at least one ACE. In comparison, the Felitti et al. (1998) landmark ACE study with 8,056 predominately Caucasian (79%) college graduates (43%), had 52% reporting at least one ACE, with 6.2% reporting more four or more ACEs. Similarly, Bellis et al. (2017) had a sample of 7,047 participants (85% Caucasian) and 10% of their sample had four or more ACEs. Gilbert and colleagues (2015) presented a large sample size of 53,998 in the District of Columbia (80% Caucasian, 38% college graduates), 12.7% reported to having more than four ACEs.

**Table 3:** ACE Scores

**# of ACEs**	**N**	**%**	**M**	**SD**
0	73	33	1.72	1.91
1	58	26.2		
2	30	13.6		
3	24	10.9		
4	12	5.4		
5	10	4.5		
6	8	3.6		
7	3	1.4		
8	3	1.4		
Total	221	100		

This table represents the frequencies of ACE scores. N, %, M, SD represent total count, percent, mean and standard deviation, respectively

Additionally, findings identified potential risk factors for mental distress among Hispanic. The first is the association between being U.S born and higher mental distress. This finding is consistent with previous research, which identified higher rates of mental distress among U.S. born Hispanic and higher rates of ACEs (Caballero et al., 2017). Mental distress among U.S. born Hispanic may be due to challenges related to parental immigration status, acculturation processes, and acculturative stress (Katiria et al., 2014; Rojas-Flores et al., 2017). The second risk factor is past year threatening events, which were positively associated with mental distress. Interestingly, previous literature links parental immigration status with threatening events for Hispanic individuals, which may contribute to both depression and anxiety (Caplan et al., 2012; Chavez-Dueña et al., 2019).

### Strengths and Limitations

Our study demonstrated several strengths. First, this study was conducted on an a priori basis, with hypotheses developed first, followed by a power analysis, data collection and planned statistical tests. Second, all participants completed the entire survey, which avoided problems with bias from missing data. Third, the survey utilized established measures with known psychometric properties. The scales measuring ACEs, mental distress (Easton et al., 2017; Prochaska et al., 2012) and resilience (Dong et al., 2017; Roy et al., 2011) have been used extensively and have established reliability and validity. Our focus on a predominantly Hispanic, low-income population is also a strength as previous work on ACEs has largely been conducted with Caucasian populations.

One important limitation of this study is that, due to the cross-sectional nature of our data collection, we were not able to infer causation. Future longitudinal research that randomly assigns participants to a resilience promoting intervention or control condition can more rigorously test a causal model of resilience moderating the relation between ACEs and mental distress. A second limitation arises from the complexity of resilience as a construct, which has been defined in different ways and may vary according to culture and environment (Rutten et al., 2013; Ungar & Liebenberg, 2011; Ungar, 2013). Nevertheless, resilience is thought to be a universal construct and our operationalization is consistent with leading definitions (Ungar, 2013). A third limitation of the study is that findings may not generalize to other regions due to the unique characteristics of this study’s population. Finally, a fourth limitation is the exclusive reliance on self-reported survey data, which may artificially inflate the associations found due to a method bias.

### Future Implications

Study findings suggest a need for interventions that develop resilience among individuals exposed to ACEs. One promising example is the program 2Gen Thrive, which prevents stress and promotes resilience for at-risk low-income families by improving caregiver ability to respond to a child’s development including their emotional and behavioral needs (Woods-Jaeger et al., 2018). Further development and testing are needed to identify evidence-based resilience interventions with long-term benefits.

Policies that protect children and prevent abuse are also needed to reduce the incidence of ACEs. Conditional cash-transfer programs, where parents receive financial support on the condition, they participate in programs designed to benefit their children, have been successful in several contexts (Gross et al., 2011). Another promising approach to preventing child abuse and neglect is the CDC’s Veto Violence initiative, which provides prevention tools, trainings and resources for ACE prevention (CDC, 2019). To enhance monitoring and early detection, there is a need for service providers across settings to assess and address ACEs in terms of mental health. Trainings for physicians, educators, and the community on detecting and addressing ACEs may help in this regard.

## Conclusion

This study identifies resilience as a moderator of the relation between ACEs and mental health. Given the prevalence of ACEs as a public health concern associated with poor mental health (Felitti & De Anda, 2009), unemployment (Allem et al., 2015), substance abuse (Chandler et al., 2018) and early death (Felitti et al., 1998), resilience has important implications for prevention and health promotion. This study provides the empirical rationale for a call to action to promote resiliency in Hispanic communities, given its centrality in establishing a healthy future for children and adults exposed to adversity.
